# Factors associated with the severity of nutritional anemia during pregnancy in Shenzhen (2021–2023): a cross-sectional analysis

**DOI:** 10.3389/fpubh.2026.1849564

**Published:** 2026-07-02

**Authors:** Yonghong Lian, Mi Zhang, Kaiwu Lin, Lingyu Xu, Zhuoqun Zhou, Xuemei Gao, Xinxin Hu, Olamide Adefioye, Zihan Zhong, Linrong Chen, Zhixue Xu, Weihua Zhao, Yan Tan, Guanglei Li

**Affiliations:** 1 Shenzhen Pingshan Maternity and Child Healthcare Hospital, Shenzhen, China; 2 Guangxi University of Chinese Medicine, Nanning, China; 3 Shenzhen Technology University, Shenzhen, China; 4 Shenzhen Second People’s Hospital, Shenzhen, China; 5 Terasaki Institute for Biomedical Innovation, Los Angeles, CA, United States; 6 Shenzhen Key Laboratory of Reproductive Immunology for Peri-implantation, Shenzhen Zhongshan Institute for Reproductive Medicine and Genetics, Shenzhen Zhongshan Obstetrics & Gynecology Hospital, Shenzhen, China

**Keywords:** anemia, migrant population, pregnancy, severity, Shenzhen

## Abstract

**Background:**

Nutritional anemia during pregnancy is a major public health concern and a leading contributor to adverse maternal and neonatal outcomes. The determinants of anemia severity remain insufficiently characterized in rapidly urbanizing regions. This study aimed to identify factors associated with the severity of nutritional anemia among pregnant women in Pingshan District, Shenzhen.

**Methods:**

A retrospective cross-sectional study was conducted using data from the Shenzhen Maternal and Child Health Management Information System. A total of 4,025 pregnant women diagnosed with nutritional anemia between 2021 and 2023 with complete records were included. Anemia severity was classified based on the lowest hemoglobin concentration during pregnancy as mild or moderate-to-severe. Univariate and multivariable logistic regression analyses were performed to identify independent risk factors.

**Results:**

Among the 4,025 pregnant women included, 1,176 (29.2%) had moderate-to-severe nutritional anemia. In the fully adjusted model, migrant population status was independently associated with an increased risk of moderate-to-severe anemia compared with permanent residents (OR = 1.6, 95% CI: 1.3–1.9). Higher parity was also independently associated with greater anemia severity, with women having two or more prior deliveries exhibiting a 1.4-fold increased risk (OR = 1.4, 95% CI: 1.2–1.8). In addition, after excluding cases in which prenatal risk stratification was elevated solely due to anemia, a high prenatal risk status at the initial antenatal visit remained significantly associated with moderate-to-severe anemia (OR = 1.6, 95% CI: 1.2–2.1). Conversely, a pre-pregnancy BMI ≥ 25 kg/m^2^ was inversely associated with anemia severity (OR = 0.7, 95% CI: 0.5–1.0), while maternal age was not independently associated with anemia severity.

**Conclusion:**

In this urbanized district of Shenzhen, migrant status, higher parity, and elevated prenatal risk were independent factors associated with moderate-to-severe nutritional anemia among women diagnosed with nutritional anemia. Early anemia screening and targeted nutritional interventions for these high-risk subgroups may help reduce anemia severity and improve maternal and neonatal outcomes.

## 1 Introduction

Anemia is a pathological condition characterized by hemoglobin (Hb) levels falling below the mean for a specific population, with the core feature being impaired oxygen-carrying capacity of red blood cells, leading to insufficient tissue oxygenation (1). According to the Chinese Society of Perinatal Medicine guidelines (“Guidelines for Diagnosis and Treatment of Iron Deficiency and Iron-Deficiency Anemia in Pregnancy”), anemia during pregnancy is defined as Hb < 110 g/L and classified as mild (Hb 100–109 g/L), moderate (Hb 70–99 g/L), and severe (Hb 40–69 g/L) (2). It is essential to clarify that while the etiology of anemia in pregnancy is multifaceted—including hereditary conditions such as thalassemia, immune-mediated disorders, and malabsorption due to gastric diseases—nutritional anemia remains the most prevalent type. Nutritional anemia refers to anemia caused by insufficient intake, impaired absorption, or increased demand for iron, folic acid, or vitamin B_12_, accounting for approximately 75% of all gestational anemia cases, and is the primary focus of this study (3, 4).

Pregnancy-related anemia is one of the most common maternal health challenges globally, especially nutritional anemia, affecting approximately 40% of pregnant women and significantly increasing maternal and perinatal morbidity and mortality, thus constituting a major public health burden (5). During pregnancy, the maternal body undergoes a series of physiological adaptations, including blood volume expansion, metabolic shifts, and hormonal fluctuations, which sharply increase the demand for key nutrients such as iron, folic acid, and vitamin B_12_ (6). Among these, iron is critical for supporting fetal growth, placental development, and the expansion of maternal red blood cell mass, while folic acid and vitamin B₁₂ are vital for erythropoiesis and fetal neurological development (7–9). If dietary intake fails to meet these elevated requirements, or if factors such as gastrointestinal malabsorption or pre-pregnancy menorrhagia interfere with nutrient utilization, the risk of developing nutritional anemia and its progression to more severe stages increases significantly (10, 11).

Although global studies have identified common risk factors for anemia during pregnancy—such as low socioeconomic status, unhealthy dietary patterns, and limited access to antenatal care (10, 12)—the severity of nutrition-related anemia and its determinants vary substantially across regions, influenced by local socioeconomic development, dietary culture, healthcare system capacity, and population structure. Shenzhen, one of China’s most economically developed cities, hosts a large and highly mobile population of women of reproductive age. In Pingshan District, rapid industrial growth has increased the number of pregnant women, placing growing demands on maternal healthcare services. During 2021–2023, the overall prevalence of anemia among pregnant women in Pingshan District reached 40.33%, exceeding the national average, with nutrition-related anemia accounting for the majority of cases, highlighting both a substantial regional public health burden and the potential for prevention through targeted interventions. However, evidence specifically addressing factors associated with nutrition-related anemia severity in this district remains limited.

The biological and social rationale for examining these specific factors is as follows. Higher parity may lead to cumulative depletion of maternal iron stores across successive pregnancies, particularly when interpregnancy intervals are short or postnatal recovery is insufficient. High prenatal risk status at the initial antenatal visit may reflect underlying conditions that increase iron demand or trigger inflammation, thereby interfering with iron metabolism and erythropoiesis. Pre-pregnancy body mass index (BMI) serves as a proxy for baseline nutritional reserves; women with lower BMI may have reduced iron stores before conception, whereas those with higher BMI may have a different metabolic profile that affects anemia progression.

This study, based on retrospective clinical data of women diagnosed with nutritional anemia, aims to analyze the association of population type, gravidity, parity, pre-pregnancy BMI, and prenatal risk status with anemia severity. It is emphasized that the study findings are applicable only to women already diagnosed with anemia and cannot be directly extrapolated to the general pregnant population.

## 2 Materials and methods

### 2.1 Study population

This retrospective cross-sectional study included pregnant women who registered and received prenatal care at midwifery institutions in Pingshan District, Shenzhen, between January 1, 2021 and December 31, 2023.

#### 2.1.1 Inclusion criteria

(1) Confirmed pregnancy with regular prenatal visits during the study period;
(2) Complete personal and clinical records available in the Shenzhen Maternal and Child Health Management Information System, including residency status, age, gravidity, parity, pre-pregnancy weight, pre-pregnancy Body Mass Index (BMI), and gestational hemoglobin (Hb) levels.

#### 2.1.2 Exclusion criteria

(1) Missing key research data;
(2) Pre-existing severe underlying diseases, thalassemia, or other pathological conditions that could interfere with hemoglobin levels.

**Figure 1:**
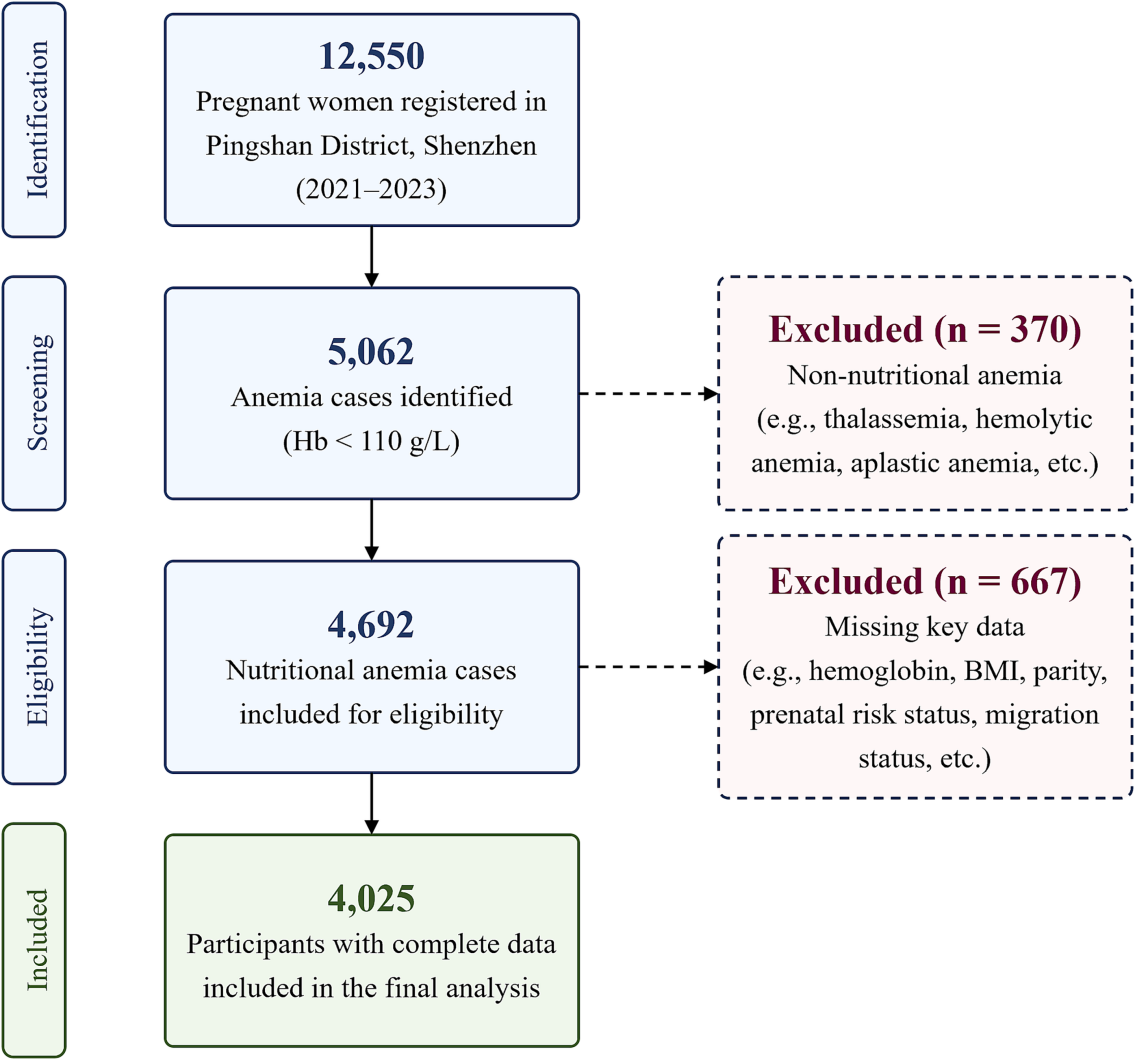
Flowchart of participant selection.

Thalassemia case identification: Cases of thalassemia were identified and excluded based on diagnostic records in the hospital information system. The system includes complete blood count and hemoglobin analysis to screen potential carriers. No systematic genetic testing was performed during the study period; therefore, residual misclassification or missed cases cannot be entirely ruled out. This potential limitation is addressed in the discussion section. A flow diagram illustrating the participant selection process is shown in Figure 1.

### 2.2 Data sources and ethics

Data were retrieved from the Shenzhen Maternal and Child Health Management Information System. The study was conducted in accordance with the Declaration of Helsinki and approved by the Medical Ethics Committee of Shenzhen Pingshan District Maternal and Child Health Hospital (Protocol Code: [2024] No. 005). Given the retrospective nature of the study and the use of de-identified, archived medical records, the requirement for informed consent was waived by the Ethics Committee. To protect participant privacy, all data were anonymized by removing personal identifiers, such as names, ID numbers, and contact information, prior to analysis.

### 2.3 Diagnostic and grading criteria

Gestational anemia was defined as an Hb concentration < 110 g/L at any point during pregnancy. Severity was classified according to the Chinese Society of Perinatal Medicine guidelines: Mild (Hb 100–109 g/L), Moderate (Hb 70–99 g/L), Severe (Hb 40–69 g/L).

### 2.4 Variables and definitions

#### 2.4.1 Outcome variable

The primary outcome was Anemia Severity, determined by the lowest recorded Hb value during pregnancy among all available prenatal test results. For statistical analysis, participants were dichotomized into two groups: the Mild Anemia group (Hb 100–109 g/L) and the Moderate-to-Severe Anemia group (Hb < 100 g/L).

#### 2.4.2 Explanatory variables

Maternal age at delivery was categorized according to commonly used obstetric clinical criteria as the following:

< 35 years.

≥35 years.

Population type was classified as:

Permanent residents.

Migrant population.

Permanent residents referred to pregnant women with Shenzhen household registration, whereas migrant population referred to those without local household registration who resided in Pingshan District and received antenatal care during the study period. In Shenzhen, pregnant women without local hukou and without stable employment cannot participate in the city’s basic medical insurance as individuals. Even when they have health insurance from their place of origin, reimbursement for out-of-town outpatient prenatal care, follow-up visits, and iron supplement prescriptions involves cumbersome procedures and limited coverage, substantially increasing out-of-pocket expenses. This variable was therefore considered a composite proxy for lower socioeconomic status and reduced healthcare access.

Pre-pregnancy body mass index (BMI) was calculated as weight (kg) divided by height squared (m^2^), and categorized according to the Chinese Guidelines for the Prevention and Control of Overweight and Obesity in Adults. For statistical analysis, due to the relatively small number of participants in the obesity group (BMI ≥ 30.0 kg/m^2^), the overweight and obesity categories were combined, resulting in three BMI groups as follows:

Underweight: < 18.5 kg/m^2^.

Normal weight: 18.5–24.9 kg/m^2^.

Overweight/obesity: ≥ 25.0 kg/m^2^.

Prenatal risk status at the initial antenatal visit was assessed based on the Maternal Pregnancy Risk Assessment Form issued by the National Health Commission of China (No. 42 [2017], Appendix 2) (13). This assessment system stratifies pregnancy risk by comprehensively evaluating pre-existing maternal conditions and abnormal findings identified at the initial antenatal visit. The evaluation includes, but is not limited to, adverse obstetric history (such as recurrent miscarriage, preterm birth, or perinatal death), uterine scarring, multiple pregnancy, and pregnancy complicated by cardiovascular diseases, endocrine disorders (e.g., diabetes mellitus and thyroid disorders), hematological diseases, or hypertensive disorders of pregnancy. Based on these assessments, pregnancy risk at the initial antenatal visit is categorized into four levels: low risk, moderate risk, relatively high risk, and high risk.

Given the limited number of participants in the relatively high- and high-risk categories, these two groups were combined into a single high-risk group for statistical analysis. Ultimately, prenatal risk was categorized into three levels:

Low risk.

Moderate risk.

High risk.

Notably, anemia itself constitutes one of the criteria in the original risk assessment system. To avoid outcome-related misclassification and potential contamination of independent variables, pregnant women whose risk level was elevated solely due to anemia, in the absence of any other positive risk indicators, were reclassified into the low-risk group.

### 2.5 Statistical analysis

Statistical analyses were performed using SPSS version 26.0 (IBM Corp., Armonk, NY, USA). Continuous variables were expressed as mean ± standard deviation (SD) or median with interquartile range (IQR), depending on their distribution. Categorical variables were presented as frequencies and percentages [*n* (%)].

Univariate analyses were conducted to assess associations between anemia severity and potential explanatory variables, including population type, maternal age, pre-pregnancy BMI, parity, and prenatal risk status at the initial antenatal visit. Variables for the multivariable binary logistic regression models were pre-selected based on a predefined theoretical framework incorporating relevant biological and social factors. Of the 4,692 pregnant women initially identified with nutritional anemia, 4,025 were included in the multivariable analysis. Excluded cases lacked essential sociodemographic information required for regression analysis. Specifically, the most frequently missing variables were pre-pregnancy BMI and migrant status, as these fields were not mandatory in the routine clinical data entry system. The missing data were random and not associated with anemia severity. All statistical tests were two-sided, and *p* < 0.05 was considered statistically significant.

## 3 Results

### 3.1 Population characteristics and prevalence trends

From 2021 to 2023, a total of 12,550 pregnant women were registered in Pingshan District, Shenzhen. Anemia was identified in 5,062 cases, constituting an overall prevalence of 40.33%. After excluding non-nutritional cases (e.g., thalassemia, immune-mediated anemia, and gastric malabsorption; *n* = 370), 4,692 cases were confirmed as nutritional anemia, with a prevalence of 37.39%. Notably, the annual prevalence of nutritional anemia exhibited a significant downward trend over the study period, decreasing from 42.96% in 2021 to 38.26% in 2022 and further to 32.25% in 2023. Among these cases, 4,025 women with complete data were included in the subsequent analysis of anemia severity.

### 3.2 Baseline characteristics of the study population and univariate analysis of factors associated with anemia severity

Table 1 summarizes the demographic and clinical characteristics of the study population stratified by anemia severity, along with the results of univariate logistic regression analyses. Among the 4,025 pregnant women included, 1,176 (29.2%) were classified as having moderate-to-severe nutritional anemia.

**Table 1:** Baseline demographic and clinical characteristics of pregnant women by anemia severity and univariate logistic regression analysis (*n* = 4,025).

Variables	Mild nutritional anemia (*n* = 2,849)	Moderate-to-severe nutritional anemia (*n* = 1,176)	OR (95%CI)	*p*
Age, years				
<35	2,447 (85.9%)	993 (84.4%)	ref	
≥35	402 (14.1%)	183 (15.6%)	1.1 (0.9, 1.4)	0.235
Population type				
Permanent resident	596 (20.9%)	167 (14.2%)	ref	
Migrant population	2,253 (79.1%)	1,009 (85.8%)	1.6 (1.3, 1.9)	<0.001***
Prenatal risk status at initial antenatal visit				
Low risk	1,491 (52.3%)	557 (47.4%)	ref	
Moderate risk	1,160 (40.7%)	498 (42.3%)	1.1 (1.0, 1.3)	0.057
High risk	198 (6.9%)	121 (10.3%)	1.6 (1.3, 2.1)	<0.001***
Gravidity, *n*				
1	753 (26.4%)	275 (23.4%)	ref	
2	904 (31.7%)	361 (30.7%)	1.1 (0.9, 1.3)	0.342
>2	1,192 (41.8%)	540 (45.9%)	1.2 (1.0, 1.5)	0.014*
Parity, *n*				
0	1,053 (37.0%)	359 (30.5%)	ref	
1	1,324 (46.5%)	563 (47.9%)	1.2 (1.1, 1.5)	0.005**
>1	472 (16.6%)	254 (21.6%)	1.6 (1.3, 1.9)	<0.001***
Pre-pregnancy BMI, kg/m^2^				
<18.5	410 (14.4%)	194 (16.5%)	ref	
18.5 ~ 25	2,129 (74.7%)	852 (72.4%)	0.8 (0.7, 1.0)	0.081
≥25	310 (10.9%)	130 (11.1%)	0.9 (0.7, 1.2)	0.375

*p < 0.05, **p < 0.01, ***p < 0.001.

Univariate analysis demonstrated a significant association between population type and increased anemia severity. Compared with permanent residents, migrant women had 1.6-fold increased odds of developing moderate-to-severe anemia (OR = 1.6; 95% CI: 1.3–1.9; *p* < 0.001). Obstetric history was also significantly associated with anemia severity. Women with more than two pregnancies showed a modestly increased odds of moderate-to-severe anemia (OR = 1.2; 95% CI: 1.0–1.5; *p* = 0.014), while those with more than one prior delivery exhibited a substantially higher risk (OR = 1.6; 95% CI: 1.3–1.9; *p* < 0.001), compared with their respective reference groups.

In addition, prenatal risk status at the initial antenatal visit was positively associated with anemia severity. Women classified as high-risk had significantly higher odds of moderate-to-severe anemia than those in the low-risk group (OR = 1.6; 95% CI: 1.3–2.1; *p* < 0.001). By contrast, maternal age (≥35 years) was not significantly associated with anemia severity in the univariate model (*p* = 0.235). Similarly, pre-pregnancy BMI showed no statistically significant association with moderate-to-severe anemia, although women with normal BMI tended to have a lower odds compared with underweight women (*p* = 0.081).

### 3.3 Multivariable logistic regression analysis of factors associated with anemia severity

To further identify independent factors associated with the severity of nutritional anemia, multivariable logistic regression models were constructed (Table 2). After full adjustment for potential confounders (Adjust II), migrant population status, parity, pre-pregnancy BMI, and prenatal risk status at the initial antenatal visit remained significantly associated with moderate-to-severe nutritional anemia.

**Table 2:** Multivariable logistic regression analysis of factors associated with moderate-to-severe nutritional anemia.

Variable	Adjust I OR (95%CI)	*p*	Adjust II OR (95%CI)	*p*
Age, year				
<35	Reference			
≥35	1.1 (0.9, 1.5)	0.278	1.0 (0.8, 1.2)	0.913
Population type				
Permanent resident	Reference			
Migrant population	1.7 (1.4, 2.0)	<0.001***	1.6 (1.3, 1.9)	<0.001***
Prenatal risk status at initial antenatal visit				
Low risk	Reference			
Moderate risk	1.2 (1.0, 1.3)	0.048*	1.1 (0.9, 1.3)	0.456
High risk	1.7 (1.3, 2.2)	<0.001***	1.6 (1.2, 2.1)	0.003**
Parity, *n*				
0	Reference			
1	1.3 (1.1, 1.5)	0.002**	1.3 (1.1, 1.5)	0.005**
>1	1.7 (1.4, 2.1)	<0.001***	1.4 (1.2, 1.8)	<0.001***
Pre-pregnancy BMI, kg/m^2^				
<18.5	Reference			
18.5 ~ 25	0.8 (0.7, 1.0)	0.064	0.9 (0.7, 1.1)	0.159
≥25	0.9 (0.7, 1.1)	0.313	0.7 (0.5, 1.0)	0.032*

Adjust I model was adjusted for age.

Adjust II model was adjusted for age, population type, prenatal risk status at initial antenatal visit, parity, and pre-pregnancy BMI.

*p < 0.05, **p < 0.01, ***p < 0.001.

In the fully adjusted model, migrant population status was significantly associated with an increased likelihood of moderate-to-severe anemia. Compared with permanent residents, migrant women had 1.6-fold increased odds of developing moderate-to-severe anemia (OR = 1.6; 95% CI: 1.3–1.9; *p* < 0.001). With respect to obstetric history, parity showed a clear positive association with anemia severity. Women with one prior delivery had 1.3-fold increased odds (OR = 1.3; 95% CI: 1.1–1.5; *p* = 0.005), while those with more than one prior delivery had 1.4-fold increased odds of moderate-to-severe anemia compared with nulliparous women (OR = 1.4; 95% CI: 1.2–1.8; *p* < 0.001).

Additionally, women classified as having a high prenatal risk status at the initial antenatal visit were significantly more likely to develop moderate-to-severe anemia than those in the low-risk group (OR = 1.6; 95% CI: 1.2–2.1; *p* = 0.003), whereas no statistically significant association was observed for the moderate-risk group in the fully adjusted model.

Notably, after adjustment for all covariates, a pre-pregnancy BMI ≥ 25 kg/m^2^ was inversely associated with anemia severity, indicating a protective association against moderate-to-severe nutritional anemia (OR = 0.7; 95% CI: 0.5–1.0; *p* = 0.032). In contrast, maternal age was not independently associated with anemia severity in the multivariable analysis (*p* = 0.913).

## 4 Discussion

In this population-based study, we analyzed clinical data from 4,025 pregnant women diagnosed with nutritional anemia in Pingshan District, Shenzhen, between January 2021 and December 2023. The primary objective was to identify factors associated with the severity of nutritional anemia during pregnancy, thereby providing evidence to inform targeted prevention and management strategies for maternal anemia in this district.

### 4.1 Regional prevalence trends and the thalassemia context

From a regional perspective, the overall anemia prevalence of 40.33% and nutritional anemia prevalence of 37.39% in Pingshan District are higher than the national average (30.7%) and the prevalence reported in Northwest China (34.8%), while remaining consistent with findings from Guangzhou (38.8%) (14–16). This elevated prevalence may be partly explained by the unique demographic composition of Pingshan, a rapidly urbanizing core district in the Guangdong-Hong Kong-Macao Greater Bay Area, which hosts a high proportion of migrant populations. These populations may face challenges in healthcare accessibility and adherence to nutritional interventions, which are associated with higher localized anemia prevalence.

Notably, the annual decline in nutritional anemia prevalence from 2021 to 2023 likely reflects improvements in maternal healthcare, including pre-pregnancy education, standardized antenatal care, and routine iron supplementation. This is consistent with Zhou et al.’s conclusion that standardized screening and targeted interventions can effectively reduce the anemia burden, indicating that current measures are effective but may require further optimization for high-risk groups.

Furthermore, given that the carrier rate of thalassemia genes in Guangdong is as high as 19.48% (17), the 370 cases identified in this study (7.31% of the total anemic population) align with the regional characteristics of high thalassemia prevalence. While the 19.48% figure refers to the general population (including asymptomatic carriers), the 7.31% in our study represents a subset of anemic patients, highlighting the objective presence of thalassemia in Pingshan.

These findings underscore the need for a dual strategy: integrating nutritional interventions with thalassemia screening. It is crucial to distinguish the additive effect of iron deficiency and thalassemia in order to prevent progression of anemia severity and to avoid inappropriate iron supplementation in patients whose anemia is primarily due to hemoglobinopathies rather than nutrient deficiency.

### 4.2 Maternal age and anemia severity

In this study, maternal age was not independently associated with the severity of nutritional anemia. Women aged ≥35 years did not show a significantly increased risk of moderate-to-severe anemia compared with younger women (OR = 1.0; 95% CI: 0.8–1.2; *p* = 0.913), consistent with the univariate analysis (*p* = 0.235).

Previous studies have reported mixed results regarding the influence of advanced maternal age on anemia risk. Some have suggested that older pregnant women may be more susceptible due to cumulative nutritional depletion or comorbidities (18), while others found no significant association (19). In the present study, the lack of association may reflect the relatively young age distribution and high coverage of routine prenatal care, which could mitigate potential age-related risks.

These findings suggest that maternal age alone may not be a major determinant of nutritional anemia severity in well-supported urban populations. However, it remains important to consider age in combination with other social and obstetric factors when evaluating risk.

### 4.3 Impact of residency status and socioeconomic factors

In this study, residency status was independently associated with the severity of nutritional anemia. After full adjustment for confounders, migrant women had a significantly higher likelihood of moderate-to-severe anemia than permanent residents (OR = 1.6; 95% CI: 1.3–1.9; *p* < 0.001), indicating social context influences disease severity beyond clinical risk factors.

Residency status may reflect broader socioeconomic determinants that were not directly measured in this study. These include income level, employment stability, access to comprehensive health insurance, and dietary conditions, which have previously been shown to be associated with maternal anemia risk and severity (15, 20). For example, national surveys in China have shown that lower socioeconomic status and limited access to antenatal services are associated with higher anemia prevalence among pregnant women.

Although our dataset lacked detailed measures of these socioeconomic variables, the strong association between migrant status and anemia severity suggests that interventions targeting social determinants—in addition to clinical care—are important for reducing anemia burden among highly mobile urban populations.

From a health policy perspective, during the study period (2021–2023)—which preceded the implementation of Shenzhen’s new medical insurance regulations on October 1, 2023—migrant women without local hukou and stable employment faced substantial barriers to accessing timely antenatal care. They were ineligible to enroll in Shenzhen’s basic medical insurance as individuals, and reimbursement for out-of-town outpatient services was administratively complex with limited coverage, leading to higher self-financed expenditures. These financial and administrative obstacles likely reduced adherence to scheduled prenatal visits and prescribed iron supplementation, thereby contributing to the observed association between migrant status and greater anemia severity.

### 4.4 Pregnancy risk assessment and pathophysiological mechanisms

Another significant result is the inverse correlation between initial pregnancy risk assessment and Hb levels. Higher risk levels were associated with greater anemia severity (adjusted OR for high risk = 1.6, *p* = 0.003), indicating that high-risk pregnancies are more susceptible to severe anemia. Based on previous studies, high-risk pregnancies may be associated with increased metabolic demand for iron and other micronutrients, while underlying comorbidities may contribute to impaired nutrient absorption (21). The total additional iron requirement during pregnancy is approximately 1 g, and high-risk conditions—driven by metabolic disorders or inflammation—can increase this demand by 40–60%. For instance, Georgieff noted that gestational diabetes involves insulin-resistance-related iron utilization disorders, and pregnancy-induced hypertension involves inflammation-mediated hepcidin elevation that inhibits iron absorption (22). In Pingshan, incorporating nutritional assessment into the management of high-risk pregnancies is essential for early identification and personalized intervention.

### 4.5 Parity and cumulative depletion of maternal iron stores

Analyses of obstetric history revealed an independent association between parity and the severity of nutritional anemia. After adjusting for potential confounders, women with two or more prior deliveries had a significantly higher risk of moderate-to-severe anemia (OR = 1.4; 95% CI: 1.2–1.8; *p* < 0.001). This finding indicates that successive pregnancies may lead to cumulative depletion of maternal iron stores, particularly when interpregnancy intervals are short or postnatal nutritional recovery is insufficient. Although some studies have not observed a significant association, discrepancies may be explained by differences in sample size, population characteristics, or healthcare access (23–25). In highly urbanized areas such as Pingshan, multiparous women—especially among migrant populations—represent a high-risk subgroup that warrants enhanced nutritional monitoring and targeted interventions to prevent progression to moderate-to-severe anemia.

### 4.6 Pre-pregnancy body mass index and anemia severity

An inverse association was observed between pre-pregnancy body mass index and the severity of nutritional anemia. In the fully adjusted model, women with BMI ≥ 25 kg/m^2^ had a lower risk of moderate-to-severe anemia (OR = 0.7; 95% CI: 0.5–1.0; *p* = 0.032), suggesting a potential protective effect.

This observation is consistent with findings from Bodnar et al., who reported no positive association between higher BMI and gestational anemia severity, despite an increased risk of postpartum anemia (26). In the specific sociodemographic context of Pingshan District, women with higher BMI may have relatively greater overall nutritional intake or receive more intensive antenatal monitoring, which could mitigate the progression of anemia. It should also be noted that this inverse association may partly reflect the fact that our analysis was restricted to women already diagnosed with nutritional anemia; therefore, these findings should not be interpreted as evidence that higher BMI protects against anemia in the general pregnant population. However, given the borderline statistical significance and observational design, these results should be interpreted with caution, and confirmation in prospective studies is warranted.

### 4.7 Limitations

Several limitations should be noted. First, the cross-sectional design limits causal inference between the identified factors and anemia severity; longitudinal studies tracking hemoglobin levels and adherence to nutritional interventions are warranted.

Second, this study was conducted in Pingshan District, Shenzhen, and may not fully generalize to regions with different demographic profiles or healthcare systems. However, as a rapidly urbanizing district with substantial population mobility, the findings remain relevant for similar urban settings.

Third, a portion of cases (*n* = 667) were excluded from multivariable analysis due to missing sociodemographic covariates, which were mostly random and unlikely to bias the main results.

Fourth, the maternal and child health management system lacked detailed information on dietary intake, iron supplementation adherence, household income, and inflammatory markers. Cases of thalassemia or other hemoglobinopathies could not be fully excluded via genetic screening. Although no confirmed cases of megaloblastic anemia were observed, other rare subtypes of nutritional anemia cannot be entirely excluded. Additionally, the gestational age at which the lowest hemoglobin level was identified was not recorded in the dataset, which precludes a more precise assessment of the timing of anemia.

Future multicenter and prospective studies incorporating more detailed nutritional, socioeconomic, and biochemical data are needed to validate and extend these findings.

## 5 Conclusion

This study identifies migrant population status, higher parity, and high prenatal risk at the initial antenatal visit as independent factors associated with moderate-to-severe nutritional anemia in pregnant women in an urbanized district of Shenzhen. Pre-pregnancy BMI ≥ 25 kg/m^2^ was inversely associated with anemia severity, while maternal age showed no independent effect.

These findings highlight the need for targeted nutritional interventions, early anemia screening, and individualized iron supplementation for high-risk and migrant pregnant women, as well as careful differentiation between nutritional anemia and thalassemia, to reduce the burden of moderate-to-severe anemia and improve maternal and neonatal outcomes.

## Data Availability

The raw data supporting the conclusions of this article will be made available by the authors, without undue reservation.
